# Response to “Clinical recommendations for use of lidocaine lubricant during bowel care after spinal cord injury prolong care routines and worsen autonomic dysreflexia: results from a randomized clinical trial” – the authors reply

**DOI:** 10.1038/s41393-021-00716-3

**Published:** 2021-10-22

**Authors:** Vera-Ellen M. Lucci, Maureen S. McGrath, Jessica A. Inskip, Shirromi Sarveswaran, Rhonda Willms, Victoria E. Claydon

**Affiliations:** 1 Department of Biomedical Physiology and Kinesiology, Simon Fraser University, Burnaby, BC Canada; 2 International Collaboration on Repair Discoveries (ICORD), University of British Columbia, Vancouver, BC Canada; 3 GF Strong Rehabilitation Centre, Spinal Cord Injury Program, Vancouver Coastal Health, Vancouver, BC Canada; 4 Division of Physical Medicine and Rehabilitation, Faculty of Medicine, UBC, Kelowna, BC Canada

**Keywords:** Quality of life, Hypertension

## To the Editor

We thank Gray et al. [1] for their interest in our study examining the utility of topical lidocaine lubricant for the amelioration of autonomic dysreflexia (AD) during bowel care in individuals living with spinal cord injury (SCI) [2]. We are pleased they found the work interesting, rigorous, clinically-relevant, and to be an important contribution to the field. We are also pleased they recognized the strengths of the study design, use of state-of-the-art monitoring, and real-world focus, examining the at-home experiences of bowel care for people with SCI. We agree that bowel care and the burden of associated AD are important issues impacting quality of life for individuals with SCI.

Gray et al., questioned whether topical lidocaine failed to improve AD during bowel care, based on concerns that the primary outcome was the peak systolic arterial pressure (SAP) and not the change in SAP relative to baseline, which they argue is more compatible with the current Paralyzed Veterans Association (PVA) Clinical Practice guidelines definition [1]. We were concerned that expressing responses relative to baseline might be problematic given that any AD present during the baseline reading could not be standardized (by definition participants had the potential for AD at baseline because they had not yet completed their bowel care and likely had a distended bowel). There is also insufficient data regarding whether the true risk of AD is related to how much blood pressure increases, how high blood pressure becomes, or simply whether AD is present or not. For these reasons we used the peak blood pressure as the primary outcome measure, and the change relative to baseline and incidence of AD as secondary outcomes. Using all metrics of AD classification, lidocaine did not provide benefit in terms of severity of AD during bowel care. Of note, Gray et al. assert that we did not report the incidence of AD. This is incorrect; we used the PVA definition of AD (increase in SAP > 20 mmHg from baseline) and showed that all participants experienced AD during both placebo and lidocaine arms of the study – in no participant was AD prevented with lidocaine, and the magnitude of the blood pressure rise was not blunted with lidocaine. Furthermore, our data showed that the peak SAP was significantly higher, and the overall AD burden was significantly worse with lidocaine use. Accordingly, by every metric, the incidence and severity of AD was not improved with lidocaine use, in fact blood pressure was higher for longer with lidocaine.

Not only was lidocaine ineffective at ameliorating AD, it also had the unfortunate side effect of impairing reflexive defecation and bowel emptying, reflected in an increased time to complete bowel care - one participant was not able to pass stool when using lidocaine.

Gray et al., questioned our statistical approach regarding the analysis of cardiac arrhythmia during bowel care. Our data showed that the number of all-cause arrhythmic events during bowel care was greater with lidocaine than placebo (*p* = 0.011). Sub-analyses based on the source of arrhythmic activity did not quite achieve criteria for statistical significance as the authors note, with *p* values just exceeding our threshold. The terminology for a “trend” in these sub-analyses is not ideal, however, we reported exact *p* values to aid data interpretation. The statistical significance of the overall incidence of arrhythmia, regardless of location in the conducting system, was robust. Of note, the study sample size for this study, while small, met our sample size calculation for the primary outcome measure (the peak SAP) with adequate retrospective power. Given the detrimental effect of lidocaine observed we felt it was not ethically appropriate to continue recruitment beyond the initial target – additional trial participants would have little expectation of benefit and could potentially be exposed to increased risk through participation in the study.

Gray et al., reference the only study showing benefit of lidocaine use for bowel procedures [3], but fail to note that this study examined the use of *injected lidocaine anal block* to ameliorate AD during anorectal procedures rather than topical lidocaine use (injected lidocaine is not feasible for routine at-home care). They also did not examine the impact on AD during routine bowel care, ability to successfully complete bowel care, or incidence of associated cardiac arrhythmia. In fact, a different study from the same research group also reported that *topical* lidocaine did not significantly limit or prevent AD during anorectal procedures [4]. The emphasis on testing the impact of interventions on the severity of AD during routine bowel care rather than anorectal procedures is important because, based on our data, lidocaine impairs reflex defecation, resulting in longer and more difficult care procedures that provoke more severe AD. Clearly, studies examining AD responses to controlled bowel stimuli, where defecation is not the outcome, do not accurately reflect the reality for people living with SCI conducting personal care routines.

We disagree that these data are insufficient to question current recommendations for lidocaine use during at-home bowel care. Lidocaine hampered care routines and impaired defecation with no improvement in AD by any metric (including that recommended by Gray et al.), and worse AD by several measures, with a concurrent increase in all-cause cardiac arrhythmia. We believe these robust findings should not be dismissed based on minor technicalities in reporting or preferences in primary outcome measures. Improvements to bowel care are prioritized by people with SCI, with amelioration of associated AD and reductions in the time taken to complete bowel care identified as priorities to improve quality of life [5]. Lidocaine use during routine bowel care did not improve either of these metrics, and in fact worsened AD and increased time to complete care. Lidocaine lubricants should not be recommended for routine bowel care in individuals with SCI.
